# Supplementary material for: DOMINO: a network‐based active module identification algorithm with reduced rate of false calls
Source: Mol Syst Biol. 2021 Jan 20;17(1):e9593. doi: 10.15252/msb.20209593 (PMC7816759; doi:10.15252/msb.20209593)
Supplement: Supplementary file 1 — Appendix [file MSB-17-e9593-s001.docx]

**Appendix**

| **AMI tools - execution details…………………………………………………………………………...**  **Analyzing the network contribution to non-specific GO enrichment bias……………………………**  **Appendix Table S1. AMI algorithms included in our analysis………………………………………...**  **Appendix Table S2. The ten gene expression datasets used in our benchmark analysis. ……………**  **Appendix Table S3. The ten GWAS datasets used in our benchmark analysis. ………………………**  **Appendix Table S4. Runtimes on the original GE datasets (in seconds) …………………………….**  **Appendix Table S5. Runtimes on the original GWAS datasets (in seconds) …………………………**  **Appendix Table S6. Runtimes on ten permutations of GE and GWAS datasets (in seconds) ……….**  **Appendix Table S7. Association analysis between net-terms and rejected terms in GE datasets...….**  **Appendix Table S8. Association analysis between net-terms and rejected terms in GWAS datasets…**  **Appendix Figure S1. Summary statistics of the solutions obtained on the GE datasets…………….**  **Appendix Figure S2. Summary statistics of the solutions obtained on the GWAS datasets…………**  **Appendix Figure S3. Module-level evaluation criteria…………………………………………….....**  **Appendix Figure S4. The fraction of non-empty solutions as a function of the subsampling fraction**  **Appendix Figure S5. Comparison of the GO terms identified by each benchmarked algorithm to those identified by using the network only (net-terms) ………………………………………………………**  **Appendix Figure S6. Comparison of rejection ratios of GO terms across algorithms. ………………**  **References………………………………………………………………………………………………** | **2**  **3**  **5**  **6**  **7**  **7**  **8**  **8**  **9**  **11**  **12**  **13**  **14**  **15**  **_**  **16**  **17**  **18** |
| --- | --- |
|  |  |

**AMI tools - execution details**

The AMI algorithms that we tested differ in preprocessing, input and output. We describe below the specific execution details for each algorithm.

**jActiveModules** (Ideker *et al*, 2002). jActiveModules was written as a plugin for Cytoscape (Shannon *et al*, 2003), a powerful platform for network analysis of biological data. We modified the codebase of jActiveModules so we could run it independently of Cytoscape. jActiveModules expects a list of genes and their p-values as the gene activity scores. We increased the default number of requested modules (from n=5 to n=50) to retrieve more modules and required that reported modules would be mutually exclusive. The algorithm typically produced no more than 10 modules with more than 3 genes.

**NetBox** (Cerami *et al*, 2010). We modified NetBox codebase so we can choose the networks it uses. NetBox gets as an input a list of mutated genes, that is, binary gene activity scores. We used the genes’ q-values and set the gene score to 1 if its q-value was < 0.05, and 0 otherwise.

**BioNet** (Beisser *et al*, 2010)**.** BioNet is designed to retrieve only one module. To retrieve multiple mutually exclusive modules we executed BioNet iteratively, removing the genes in the identified module in each iteration. We stopped these iterations after retrieving modules smaller than four genes in five consecutive runs.

**HotNet2** (Leiserson *et al*, 2015)**.** HotNet2 expects gene activity scores that are calculated by mutation rate p-values (e.g., using MutSig). We transformed the q-values calculated from our datasets into $-log10(q\_value)$ scale and used them as the input activity scores. As HotNet2 execution time was longer than the other algorithms we generated only 1000 permutations for its background distribution.

**KeyPathwayMiner** (Baumbach *et al*, 2012)**.** We used the version of KPM with the greedy strategy. It expects binary gene activity scores: 1 marks a gene as active and 0 otherwise. We used the genes’ q-values and scored a gene with 1 if its q-value was < 0.05, and 0 otherwise. As the reported modules considerably overlap each other, we executed the algorithm iteratively, removing in each iteration the genes in the identified module.

**DOMINO**. DOMINO gets as an input binary gene activity scores. We used the genes’ q-values and set the gene score to 1 if its q-value was < 0.05, and 0 otherwise.

**Analyzing the network contribution to non-specific GO enrichment bias**

Since the structure of the input network can potentially be a source for the bias we detected in AMI solutions, we sought to identify GO terms that are enriched on modules detected on the network only, without considering any gene scores based on specific omics data. We wished to examine if excluding such terms could serve as an effective alternative to EMP in removing non-specific terms reported by AMI methods.

To this goal we identified modules in the DIP network using a network-based module identification algorithm (that is, an algorithm that finds modules based on network structure only, without considering activity scores). We chose the algorithm denoted as ’M1’ in the module detection DREAM challenge (Choobdar *et al*, 2019). M1 was a leading performer in the challenge and is also implemented as a convenient software.

We extracted GO terms that were enriched ($q-value<{10}^{-3}$) on these network-based modules (here too, using the entire set of network genes as the background set). Overall, 2,450 out of 6,573 (37%) BP GO terms were detected by this analysis, and we refer to them as *net-terms*. We found that the net-terms were significantly over-represented among GO terms reported by most AMI algorithms on the GE and GWAS datasets (**Figure 7 A, B**). This is expected, since connectivity is a feature sought by both community detection and AMI methods. Over-representation increased with the number of different datasets on which the GO term was detected (**Appendix Figure S5 A, B).** For example, GO terms that were called on all 10 datasets were almost exclusively net-terms.

Next, we examined whether net-terms were over-represented among the terms that failed the empirical validation. We first computed, for each algorithm and term $\tau$, the fraction of datasets in which the term appeared as significant but was not empirically validated. Let $\# EV (\tau)$ be the number of datasets in which $\tau$ appeared as empirically validated. Let $\# non-EV (\tau)$ be the number of datasets in which $\tau$ appeared as significant but was rejected, i.e., not empirically validated. Then we compute the following *rejection ratio* $\rho(\tau)$ :

$\rho(\tau)=\frac{\# non-EV (\tau)}{\# non-EV \left( \tau\right) + \#EV (\tau)}$.

Terms with high rejection $\mathrm{ratio}$are those that frequently appear as significant and fail the empirical test, possibly due to the network structure. Of course, this number should be considered alongside the value of the denominator, which counts how frequently $\tau$ appeared across datasets. In the following we considered only terms that were enriched in at least four datasets.

**Appendix Figure S6 A** and **B** show for each algorithm the distribution of rejection ratios across GO terms. As can be seen, DOMINO has substantially lower rejection ratios. The same, albeit to a lesser extent, is true for NetBox.

We then examined whether network structure is a key source for rejected terms (**Figure 7 C, D**). For each algorithm, we compared the rejection ratio of net-terms and other terms. Notably, net-terms did not show significantly high rejection ratios, except for DOMINO on GE, where it was marginally significant (p=0.02).

Last, we examined the association between net-terms and rejected terms in each solution. We summarized the results of each algorithm on each dataset in a contingency table, computed the enrichment factor (EF) for rejected terms among net-terms, and calculated p-value using the Fisher’s exact test, and corrected the results of each algorithm for multiple testing using FDR (**Appendix** **Tables S7-S8).** Most solutions had EF>1, and about half the results showed significant association between net-terms and rejected terms. The overall magnitude of the association was rather mild (mean q-value = 0.26 for GE and 0.38 for GWAS), and many false calls involved non net-GO terms.

In summary, GO terms detected by AMI methods are enriched for net-terms. This enrichment increases with the number of datasets on which the GO term was detected. In addition, a fraction of the rejected calls were driven by network topology. However, this association was overall mild and cannot be used to effectively distinguish between GO terms that reflect true biological signals (EV terms) and non-specific ones (rejected terms). Therefore, filtering out net-terms cannot serve as an alternative to our EMP procedure.

**Appendix Table S1**. AMI algorithms included in our analysis.

| **Method name** | **Published on** | **Designed for** | **Algorithmic Approach** | **Code language** | **# citations (updated to 11/2019)** |
| --- | --- | --- | --- | --- | --- |
| jActiveModules (Ideker *et al*, 2002) | 2002 | GE | Seek high scoring subnetworks either by simulated annealing (jAM_SA) or by a greedy search (jAM_greedy) | Java | 1207 |
| NetBox (Cerami *et al*, 2010) | 2010 | Somatic Mutations | Enrichment of Perturbed neighbors, Newman-Girvan (NG) modularity score | Java, Python | 304 |
| BioNet (Beisser *et al*, 2010) | 2010 | GE | Prize collecting Steiner tree | R | 218 |
| HotNet2 (Leiserson *et al*, 2015) | 2015 | Somatic Mutations | Heat diffusion | Python | 460 |
| KeyPathwayMiner (Baumbach *et al*, 2012) | 2012 | GE | Choose modules with at most K non-perturbed genes | Java | 41 |

**Appendix Table S2**. The ten gene expression datasets used in our benchmark analysis.

| **Datasets name (acronym)** | **access to data** | **Technology** | **General description** |
| --- | --- | --- | --- |
| TNFa (Schmidt *et al*, 2015) | GSE64233 | RNA-seq | TNFa, a potent inducer of immune responses |
| HC (Elkon *et al*, 2015) | GSE67478 | RNA-seq | Hair cell from the cochlea and vestibular system, compared to non-hair cell from these inner-ear organs. |
| ROR (Bayerlová *et al*, 2017) | GSE74383 | RNA-seq | RNA-Seq profiling of estrogen-receptor-positive MCF-7 cell lines with different perturbations of non-canonical WNT signaling. Comparison was made between ROR2-overexpression and control conditions. |
| SHERA (Miano *et al*, 2018) | GSE108693 | RNA-seq | Luminal lncRNAs regulation by ERα-controlled enhancers in a ligand-independent manner in breast cancer cells. Comparison was made between ER siRNA to control siRNA |
| SHEZH (Ito *et al*, 2018) | GSE109064 | RNA-seq | Downregulation of EZH2 leads to cellular senescence with features of SASP. Comparison between control to 4d samples. |
| ERS (Kroeger *et al*, 2018) | GSE106847 | RNA-seq | ATF6 encodes a transcription factor that is activated during the Unfolded Protein Response to protect cells from ER stress. Comparison was made between ATF6-activated and control cells. |
| IEM (Hertzano *et al*, 2011) | --- | Microarray | Comparison between 2 different cell types in the inner-ear: blood cells and mesenchymal cells. |
| APO (Pulikkan *et al*, 2018) | GSE101788 | RNA-seq | Comparison between ME-1 cells (a human leukemia cell line) treated with either the AI-10-49 drug (which induces apoptosis) or DMSO (control). |
| CBX (Connelly *et al*, 2019) | GSE123689 | RNA-seq | CBX8 is a subunit of the polycomb repressive complex 1 (PRC1). This RNA-seq experiment compared CBX8-KO and control cells. |
| IFT (Forbes *et al*, 2018) | GSE107230 | RNA-seq | IFT140 is involved in the formation and maintenance of cilia. This RNA-seq experiment compared uncorrected (IFT140 compound heterozygous) and gene-corrected (IFT140 heterozygous) epithelial cells isolated from patient’s iPSC that were derived from kidney organoids. |

**Appendix Table S3.** The ten GWAS datasets used in our benchmark analysis.

| **Datasets name (acronym)** | **Trait** | **Cohort size** |
| --- | --- | --- |
| BC (Michailidou *et al*, 2017) | Breast Cancer | 228,951 |
| CHD (De Lange *et al*, 2017) | Crohn’s Disease | 59,957 |
| SCZ (Ripke *et al*, 2014) | Schizophrenia | 150,064 |
| TRI (Teslovich *et al*, 2010) | Triglycerides | ~100,000 |
| T2D (Mahajan *et al*, 2018) | Type 2 Diabetes | 898,130 |
| CAD (Nelson *et al*, 2017) | Coronary Artery Disease | 155,197 |
| BMD (Kemp *et al*, 2017) | Bone Mineral Density | 142,487 |
| Height (Allen, 2010) | Height | 183,727 |
| AF (Nielsen *et al*, 2018) | Arterial Fibrillation | 1,030,836 |
| AMD (Fritsche *et al*, 2016) | Age-related Macular Degeneration | 33,976 |

**Appendix Table S4. Runtimes on the original GE datasets (in seconds)**

| **Network** | **algorithm** | **tnfa** | **hc** | **ror** | **shera** | **shezh** | **ers** | **iem** | **apo** | **cbx** | **ift** |
| --- | --- | --- | --- | --- | --- | --- | --- | --- | --- | --- | --- |
| **DIP** | **NetBox** | 23.4 | 24.6 | 24.9 | 124.2 | 32.2 | 2706.2 | 27.1 | 1079.1 | 24.7 | 29.9 |
|  | **DOMINO *** | **0.8** | **0.9** | **0.9** | **1.1** | **0.7** | **3.5** | **1.0** | **2.1** | **0.8** | **0.8** |
|  | **jAM_Greedy** | 20.0 | 25.3 | 21.9 | 21.3 | 24.6 | 26.9 | 24.9 | 22.7 | 28.4 | 26.2 |
|  | **jAM_SA** | 368.3 | 452.6 | 658.5 | 1361.8 | 847.8 | 1010.4 | 801.8 | 915.2 | 1056.9 | 1189.2 |
|  | **Bionet** | 66.3 | 199.0 | 85.9 | 500.6 | 287.7 | 193.4 | 206.0 | 172.8 | 369.6 | 634.1 |
|  | **HotNet2 **** | 60.9 | 68.8 | 61.9 | 66.6 | 62.1 | 72.2 | 69.6 | 71.1 | 63.0 | 68.3 |
|  | **KPM** | 22.5 | 78.1 | 42.0 | 66.2 | 76.5 | 118.1 | 70.1 | 75.7 | 79.5 | 75.5 |
| **HuRI** | **NetBox** | 26.0 | 38.9 | 26.4 | 5809 | 34.6 | >5hrs | 78.1 | >5hrs | 30.2 | 43.5 |
|  | **DOMINO *** | **1.2** | **1.9** | **2.6** | **1.4** | **1.8** | **4.8** | **1.3** | **2.0** | **1.4** | **1.6** |
| **STRING** | **NetBox** | 4445.8 | >5 hrs | 76.6 | >5 hrs | >5 hrs | >5 hrs | >5 hrs | >5 hrs | >5 hrs | >5 hrs |
|  | **DOMINO *** | **4.7** | **5.3** | **4.4** | **5.4** | **8.5** | **30.5** | **5.3** | **9.0** | **3.4** | **4.3** |

Performance was measured on a 44-core, 2.2 GHz server with 792 GB of RAM

* DOMINO runtimes are for steps 1-3 of the algorithm only, excluding step 0, which is executed only once per network.

** HotNet2 was the only algorithm running on multiple cores

**Appendix Table S5. Runtimes on the original GWAS datasets (in seconds)**

| **network** | **algorithm** | **brca** | **crh** | **scz** | **tri** | **t2d** | **bmd** | **amd** | **af** | **hgt** | **cad** |
| --- | --- | --- | --- | --- | --- | --- | --- | --- | --- | --- | --- |
| **DIP** | **NetBox** | 24.7 | 23.4 | 24.4 | 23.4 | 23.6 | 179.6 | 24.0 | 24.3 | 26.4 | 23.4 |
|  | **DOMINO *** | **0.7** | **0.7** | **0.8** | **0.9** | **0.5** | **1.2** | **1.0** | **0.8** | **0.8** | **0.8** |
|  | **jAM_Greedy** | 23.3 | 21.4 | 25.0 | 24.4 | 22.8 | 21.9 | 29.6 | 20.2 | 22.5 | 22.3 |
|  | **jAM_SA** | 680.1 | 545.4 | 925.8 | 1183.6 | 1193.3 | 896.1 | 1030.4 | 758.1 | 930.6 | 508.2 |
|  | **Bionet** | 311.0 | 144.6 | 496.7 | 68.6 | 36.6 | 338.0 | 60.6 | 267.7 | 312.8 | 39.7 |
|  | **HotNet2 **** | 73.4 | 74.1 | 73.8 | 70.1 | 73.7 | 73.9 | 70.5 | 74.5 | 73.1 | 73.3 |
|  | **KPM** | 75.4 | 47.9 | 76.6 | 26.0 | 11.1 | 50.7 | 25.8 | 64.9 | 75.5 | 9.6 |
| **HuRI** | **NetBox** | 59.5 | 26.6 | 37.7 | 26.6 | 26.3 | > 5 hrs. | 26.4 | 29.2 | 37.8 | 25.9 |
|  | **DOMINO *** | **1.6** | **2.0** | **1.6** | **2.3** | **1.7** | **1.3** | **0.9** | **1.4** | **1.6** | **0.7** |
| **STRING** | **NetBox** | 940.2 | 289.5 | 573.5 | 76.9 | 75.1 | > 5 hrs. | 313.0 | 171.8 | 632.0 | 69.4 |
|  | **DOMINO *** | **2.3** | **4.5** | **2.9** | **4.7** | **1.8** | **2.0** | **4.7** | **2.6** | **2.9** | **6.6** |

Performance was measured on a 44-core, 2.2 GHz server with 792 GB of RAM

* DOMINO runtimes are for steps 1-3 of the algorithm only, excluding step 0, which is executed only once per network.

** HotNet2 was the only algorithm running on multiple cores

**Appendix Table S6. Runtimes on ten permutations of GE and GWAS datasets (in seconds)**

| **network** | **algorithm** | **omics** | **Mean runtime, excluding jobs that did not terminate in 30 min** | **Number of permutations on which jobs exceeded 30 min** | **Average runtime on the original (unpermuted) datasets, excluding jobs that did not terminate in 5 hrs** | **Number of jobs on the original datasets not terminating in 5 hrs** |
| --- | --- | --- | --- | --- | --- | --- |
| DIP | NetBox | GE | 44.31 | 0 | 409.63 | 0 |
|  | DOMINO * | GE | 0.71 | 0 | 1.25 | 0 |
|  | jAM_Greedy | GE | 17.53 | 0 | 24.21 | 0 |
|  | jAM_SA | GE | 17.37 | 0 | 866.25 | 0 |
|  | Bionet | GE | 2.95 | 0 | 271.54 | 0 |
|  | HotNet2 ** | GE | 64.37 | 0 | 66.44 | 0 |
|  | KPM | GE | 55.15 | 0 | 70.42 | 0 |
|  | NetBox | GWAS | 14.71 | 0 | 39.71 | 0 |
|  | DOMINO | GWAS | 0.60 | 0 | 0.82 | 0 |
|  | jAM_Greedy | GWAS | 17.45 | 0 | 23.33 | 0 |
|  | jAM_SA | GWAS | 17.49 | 0 | 865.16 | 0 |
|  | Bionet | GWAS | 3.00 | 0 | 207.62 | 0 |
|  | HotNet2 ** | GWAS | 76.69 | 0 | 73.03 | 0 |
|  | KPM | GWAS | 43.61 | 0 | 46.36 | 0 |
| HuRI | NetBox | GE | 25.63 | 20 | 760.86 | 2 |
|  | DOMINO * | GE | 1.37 | 0 | 1.99 | 0 |
|  | NetBox | GWAS | 13.36 | 10 | 32.87 | 1 |
|  | DOMINO * | GWAS | 1.37 | 0 | 1.5 | 0 |
| STRING | NetBox | GE | 233.62 | 36 | 3443.87 | 8 |
|  | DOMINO * | GE | 2.97 | 0 | 8.07 | 0 |
|  | NetBox | GWAS | 167.03 | 10 | 349.03 | 1 |
|  | DOMINO * | GWAS | 3.13 | 0 | 3.5 | 0 |

Performance was measured on a 44-core, 2.2 GHz server with 792 GB of RAM

* DOMINO runtimes are for steps 1-3 of the algorithm only, excluding step 0, which is executed only once per network.

** HotNet2 was the only algorithm running on multiple cores

**Appendix Table S7. Association analysis between net-terms and rejected terms in GE datasets.**

| alg. | dataset | A | B | C | D | EF | pval | qval | F |
| --- | --- | --- | --- | --- | --- | --- | --- | --- | --- |
| Bionet | apo | 657 | 13 | 259 | 10 | 1.26 | 9.01E-02 | 1.20E-01 | 0.50 |
|  | cbx | 226 | 347 | 70 | 278 | 1.36 | 4.25E-10 | 1.70E-09 |  |
|  | ers | 769 | 5 | 201 | 22 | 4.16 | 5.72E-11 | 4.57E-10 |  |
|  | hc | 344 | 28 | 169 | 32 | 1.43 | 1.70E-03 | 3.39E-03 |  |
|  | ift | 500 | 203 | 251 | 201 | 1.34 | 4.68E-08 | 1.25E-07 |  |
|  | ror | 1 | 54 | 0 | 14 | 1.27 | 7.97E-01 | 7.97E-01 |  |
|  | shera | 845 | 149 | 370 | 65 | 1 | 5.38E-01 | 6.15E-01 |  |
|  | shezh | 192 | 350 | 71 | 166 | 1.07 | 7.99E-02 | 1.20E-01 |  |
| DOMINO | cbx | 1 | 55 | 0 | 7 | 1.12 | 8.89E-01 | 8.89E-01 | 0.56 |
|  | ers | 17 | 150 | 1 | 22 | 1.08 | 3.27E-01 | 4.21E-01 |  |
|  | hc | 27 | 163 | 0 | 75 | 1.47 | 7.11E-05 | 3.20E-04 |  |
|  | iem | 27 | 188 | 1 | 85 | 1.39 | 6.50E-04 | 1.95E-03 |  |
|  | ift | 50 | 218 | 1 | 76 | 1.32 | 1.46E-05 | 1.32E-04 |  |
|  | ror | 1 | 159 | 0 | 40 | 1.25 | 8.00E-01 | 8.89E-01 |  |
|  | shera | 21 | 22 | 0 | 6 | 1.27 | 2.69E-02 | 4.85E-02 |  |
|  | shezh | 23 | 83 | 0 | 19 | 1.23 | 1.48E-02 | 3.34E-02 |  |
|  | tnfa | 6 | 224 | 0 | 59 | 1.27 | 2.51E-01 | 3.76E-01 |  |
| HotNet2 | apo | 233 | 4 | 58 | 3 | 1.4 | 1.54E-01 | 2.31E-01 | 0.33 |
|  | ers | 601 | 27 | 341 | 17 | 1.05 | 4.28E-01 | 5.14E-01 |  |
|  | ift | 341 | 81 | 140 | 28 | 0.96 | 7.96E-01 | 7.96E-01 |  |
|  | ror | 11 | 28 | 0 | 19 | 1.67 | 7.36E-03 | 2.21E-02 |  |
|  | shera | 35 | 9 | 2 | 16 | 2.64 | 7.58E-07 | 4.55E-06 |  |
|  | shezh | 21 | 60 | 0 | 11 | 1.18 | 4.76E-02 | 9.53E-02 |  |
| jAM greedy | cbx | 390 | 11 | 147 | 0 | 0.73 | 1.00E+00 | 1.00E+00 | 0.33 |
|  | iem | 618 | 2 | 207 | 9 | 4.17 | 1.49E-04 | 7.26E-04 |  |
|  | ror | 494 | 5 | 199 | 7 | 1.69 | 3.24E-02 | 6.47E-02 |  |
|  | shera | 645 | 4 | 205 | 1 | 0.95 | 7.49E-01 | 8.99E-01 |  |
|  | shezh | 300 | 222 | 68 | 68 | 1.06 | 7.16E-02 | 1.07E-01 |  |
|  | tnfa | 788 | 8 | 273 | 14 | 2.06 | 2.42E-04 | 7.26E-04 |  |
| jAM SA | apo | 373 | 21 | 41 | 30 | 2.2 | 9.19E-15 | 4.13E-14 | 0.78 |
|  | cbx | 224 | 146 | 26 | 75 | 1.36 | 3.38E-10 | 7.60E-10 |  |
|  | ers | 693 | 115 | 267 | 150 | 1.67 | 9.51E-18 | 8.56E-17 |  |
|  | hc | 198 | 390 | 37 | 241 | 1.35 | 4.78E-11 | 1.43E-10 |  |
|  | iem | 345 | 3 | 56 | 7 | 2.87 | 1.21E-04 | 1.81E-04 |  |
|  | ift | 415 | 14 | 109 | 2 | 0.9 | 8.74E-01 | 8.74E-01 |  |
|  | ror | 437 | 18 | 137 | 19 | 1.55 | 4.50E-04 | 5.79E-04 |  |
|  | shera | 436 | 118 | 64 | 15 | 0.98 | 7.28E-01 | 8.19E-01 |  |
|  | shezh | 229 | 202 | 8 | 47 | 1.2 | 2.09E-08 | 3.76E-08 |  |
| KPM | cbx | 1358 | 1 | 1583 | 16 | 7.67 | 4.31E-04 | 1.08E-03 | 0.40 |
|  | ers | 1469 | 64 | 1265 | 126 | 1.59 | 5.72E-08 | 2.86E-07 |  |
|  | ror | 260 | 260 | 102 | 128 | 1.07 | 8.86E-02 | 1.43E-01 |  |
|  | shera | 1435 | 89 | 1469 | 9 | 0.54 | 1.00E+00 | 1.00E+00 |  |
|  | tnfa | 37 | 201 | 5 | 54 | 1.11 | 1.15E-01 | 1.43E-01 |  |
| NetBox | apo | 1663 | 39 | 1378 | 12 | 0.72 | 1.00E+00 | 1.00E+00 | 0.17 |
|  | cbx | 5 | 113 | 0 | 37 | 1.33 | 2.50E-01 | 3.01E-01 |  |
|  | iem | 223 | 102 | 53 | 41 | 1.14 | 1.97E-02 | 5.90E-02 |  |
|  | ror | 7 | 319 | 0 | 93 | 1.3 | 1.70E-01 | 2.55E-01 |  |
|  | shera | 136 | 1023 | 54 | 726 | 1.24 | 2.54E-04 | 1.52E-03 |  |
|  | shezh | 9 | 265 | 0 | 110 | 1.41 | 4.61E-02 | 9.23E-02 |  |

Association analysis between net-terms and rejected terms in GE datasets. Each row represents the association for one AMI solution. Columns A, B, C, D represent the numbers in the contingency table (see below). The enrichment factor (EF column) is computed according the expression (A/(A+C))/(B/(B+D). p-values (pval column) were calculated using Fisher's exact test and corrected for multiple testing for each algorithm using Bejamini Hochberg FDR (qval column)

F is the fraction of solutions on which the association was significant

| Contingency Table | | |
| --- | --- | --- |
|  | # net terms | # other terms |
| # non-EV terms | A | C |
| # EV terms | B | D |

**Appendix Table S8. Association analysis between net-terms and rejected terms in GWAS datasets.**

| alg. | dataset | A | B | C | D | EF | pval | qval | F |
| --- | --- | --- | --- | --- | --- | --- | --- | --- | --- |
| Bionet | af | 235 | 15 | 115 | 44 | 2.68 | 1.91E-09 | 5.73E-09 | 1.00 |
|  | brca | 376 | 0 | 175 | 12 | 6800 | 1.41E-06 | 2.12E-06 |  |
|  | crh | 22 | 67 | 0 | 27 | 1.41 | 0.001451 | 1.45E-03 |  |
| DOMINO | af | 44 | 149 | 1 | 66 | 1.42 | 6.89E-06 | 4.14E-05 | 0.17 |
|  | amd | 16 | 46 | 0 | 10 | 1.22 | 0.066405 | 1.99E-01 |  |
|  | brca | 2 | 18 | 0 | 3 | 1.16 | 0.750988 | 8.44E-01 |  |
|  | crh | 1 | 64 | 0 | 12 | 1.19 | 0.844156 | 8.44E-01 |  |
|  | hgt | 4 | 57 | 0 | 24 | 1.43 | 0.257734 | 5.15E-01 |  |
|  | scz | 1 | 39 | 0 | 17 | 1.43 | 0.701754 | 8.44E-01 |  |
| HotNet2 | af | 57 | 0 | 31 | 4 | 6500 | 0.018739 | 3.75E-02 | 0.50 |
|  | bmd | 4 | 12 | 0 | 1 | 1.09 | 0.764706 | 7.65E-01 |  |
| jAM_greedy | af | 127 | 162 | 25 | 66 | 1.18 | 0.003339 | 3.34E-03 | 1.00 |
|  | brca | 144 | 9 | 38 | 18 | 2.39 | 3.44E-06 | 5.74E-06 |  |
|  | cad | 203 | 34 | 32 | 23 | 1.43 | 1.56E-05 | 1.95E-05 |  |
|  | crh | 299 | 81 | 49 | 50 | 1.39 | 2.36E-08 | 5.91E-08 |  |
|  | scz | 198 | 34 | 45 | 79 | 2.7 | 5.90E-21 | 2.95E-20 |  |
| jAM_SA | bmd | 247 | 243 | 59 | 54 | 0.99 | 0.673553 | 6.74E-01 | 0.50 |
|  | hgt | 452 | 14 | 72 | 7 | 1.28 | 0.021821 | 4.36E-02 |  |
| KPM | crh | 443 | 144 | 400 | 50 | 0.72 | 1 | 1.00E+00 | 0.50 |
|  | tri | 59 | 5 | 10 | 7 | 2.05 | 0.00233 | 4.66E-03 |  |
| NetBox | amd | 1 | 33 | 0 | 0 | 1 | 1 | 1.00E+00 | 0.00 |
|  | brca | 1 | 78 | 0 | 21 | 1.27 | 0.79 | 1.00E+00 |  |
|  | tri | 2 | 52 | 0 | 24 | 1.47 | 0.476523 | 1.00E+00 |  |

Association analysis between net-terms and rejected terms in GWAS datasets. Each row represents the association for one AMI solution. Columns A, B, C, D represent the numbers in the contingency table (see below). The enrichment factor (EF column) is computed according the expression (A/(A+C))/(B/(B+D). p-values (pval column) were calculated using Fisher's exact test and corrected for multiple testing for each algorithm using Bejamini Hochberg FDR (qval column)

F is the fraction of solutions on which the association was significant

| Contingency Table | | |
| --- | --- | --- |
|  | # net terms | # other terms |
| # non-EV terms | A | C |
| # EV terms | B | D |

**
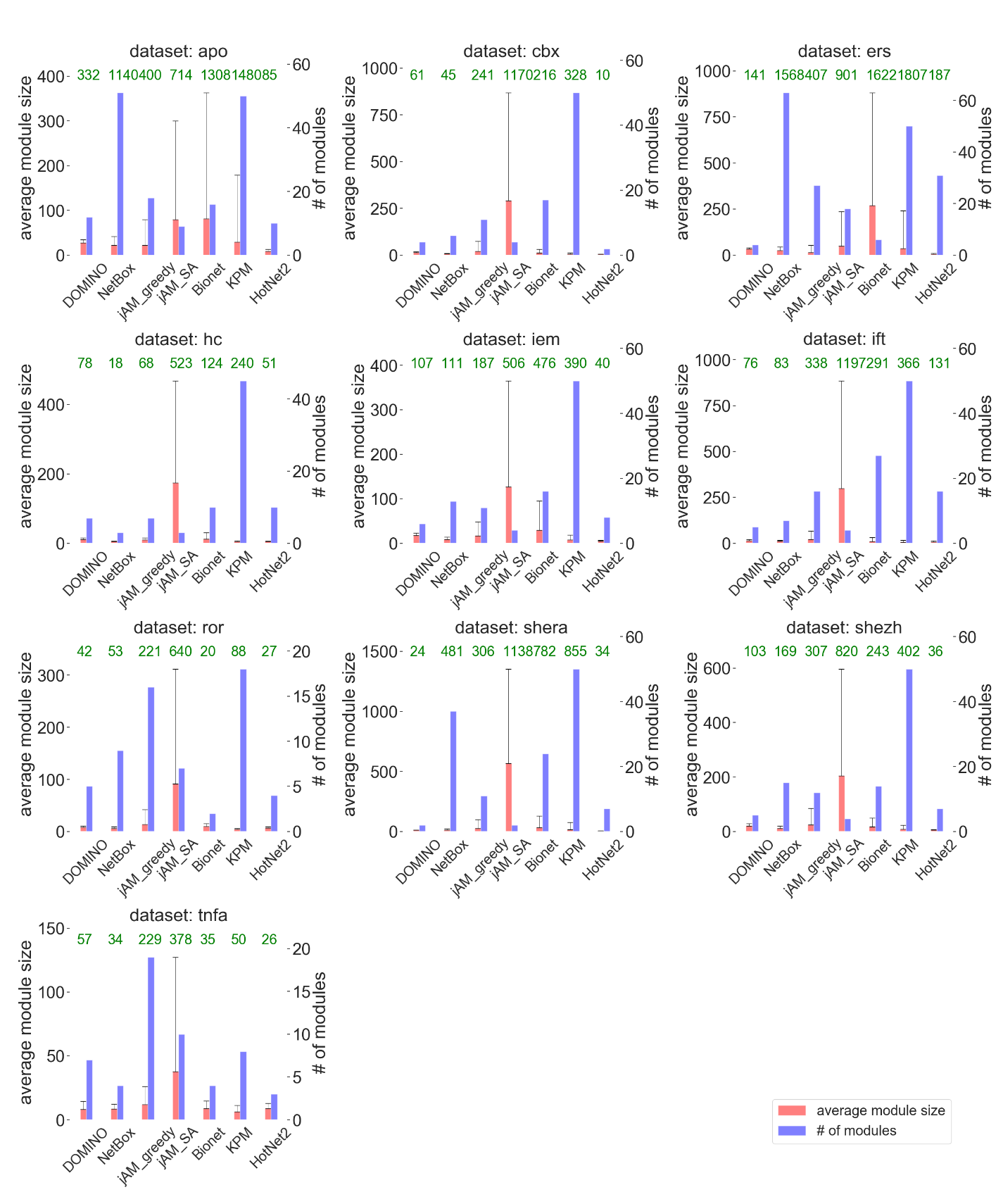
**

**Appendix Figure S1**. Summary statistics of the solutions obtained on the GE datasets. For each dataset, the number of modules detected by each AMI algorithm and their sizes are indicated. (Error bars represent 1 SD of the number of genes in modules). The numbers in green are the total number of genes in the union of all modules in the solution.

**
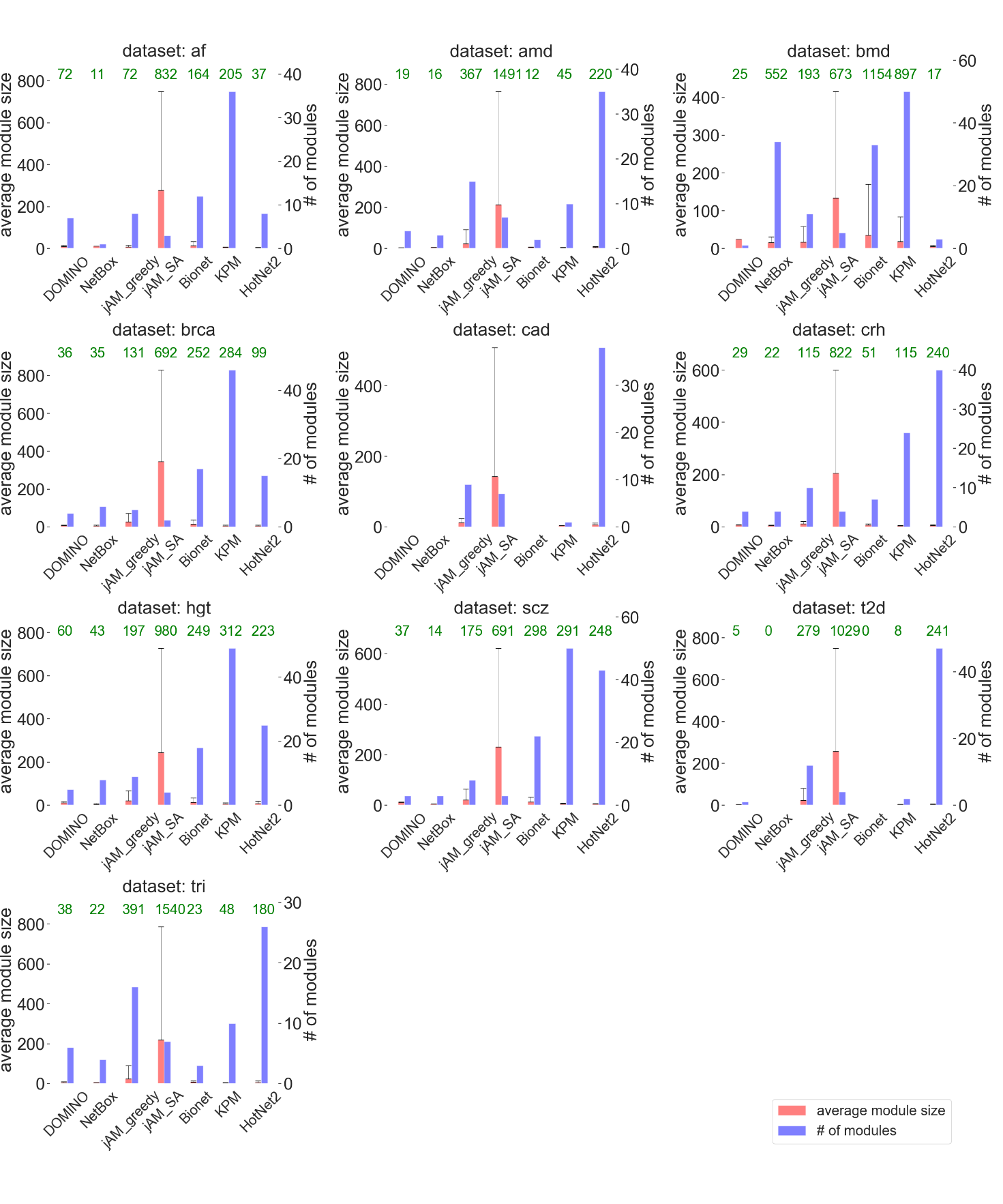
****Appendix Figure S2**. Summary statistics of the solutions obtained on the GWAS datasets. For each dataset, the number of modules detected by each AMI algorithm and their sizes are indicated. (Error bars represent 1 SD of the number of genes in modules). We excluded empty solutions. The numbers in green are the total number of genes in the union of all modules in the solution.

***Appendix Figure S3****. Module-level evaluation criteria.* ***A. mEHR****. Enriched GO terms in each module are examined by the EMP procedure (EV terms are colored in red) and mEHR is calculated for each module in the solution.* ***B.*** ***Intra-module homogeneity****. A GO graph is first built for the union of all the EV terms in a solution using Resnik similarity scores. Then, a certain cut-off is applied (here, 4.0) for filtering low scoring edges. Last, the intra-module homogeneity score is calculated as the density ratio between the EV terms that are enriched in the module and the entire GO graph.*


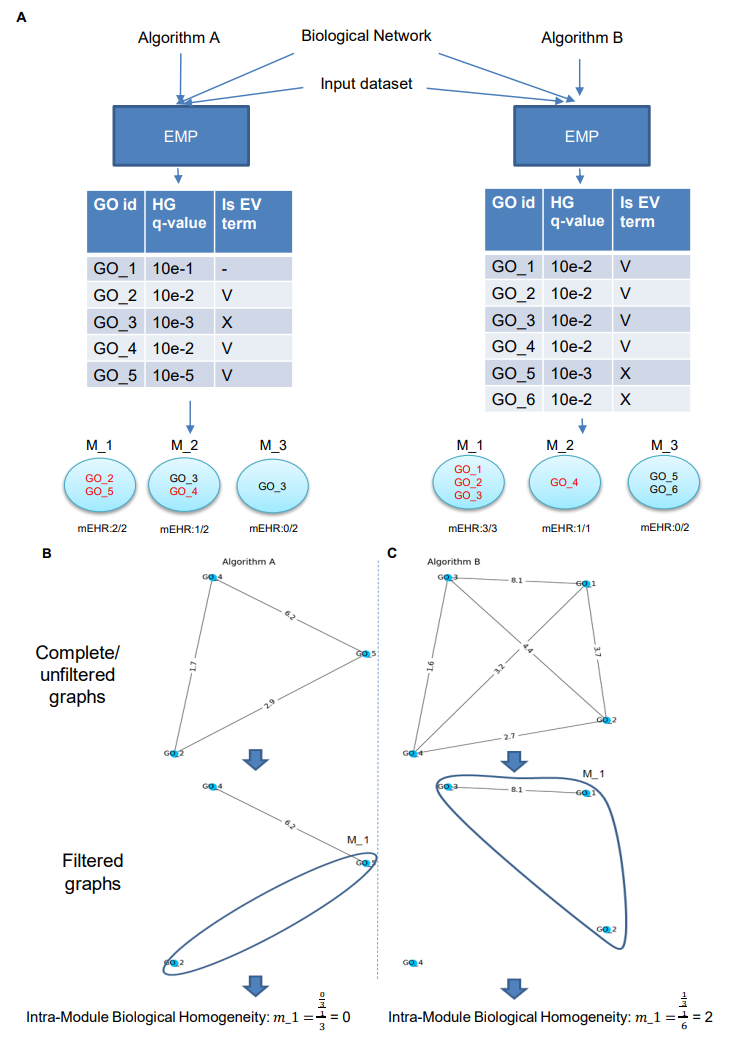

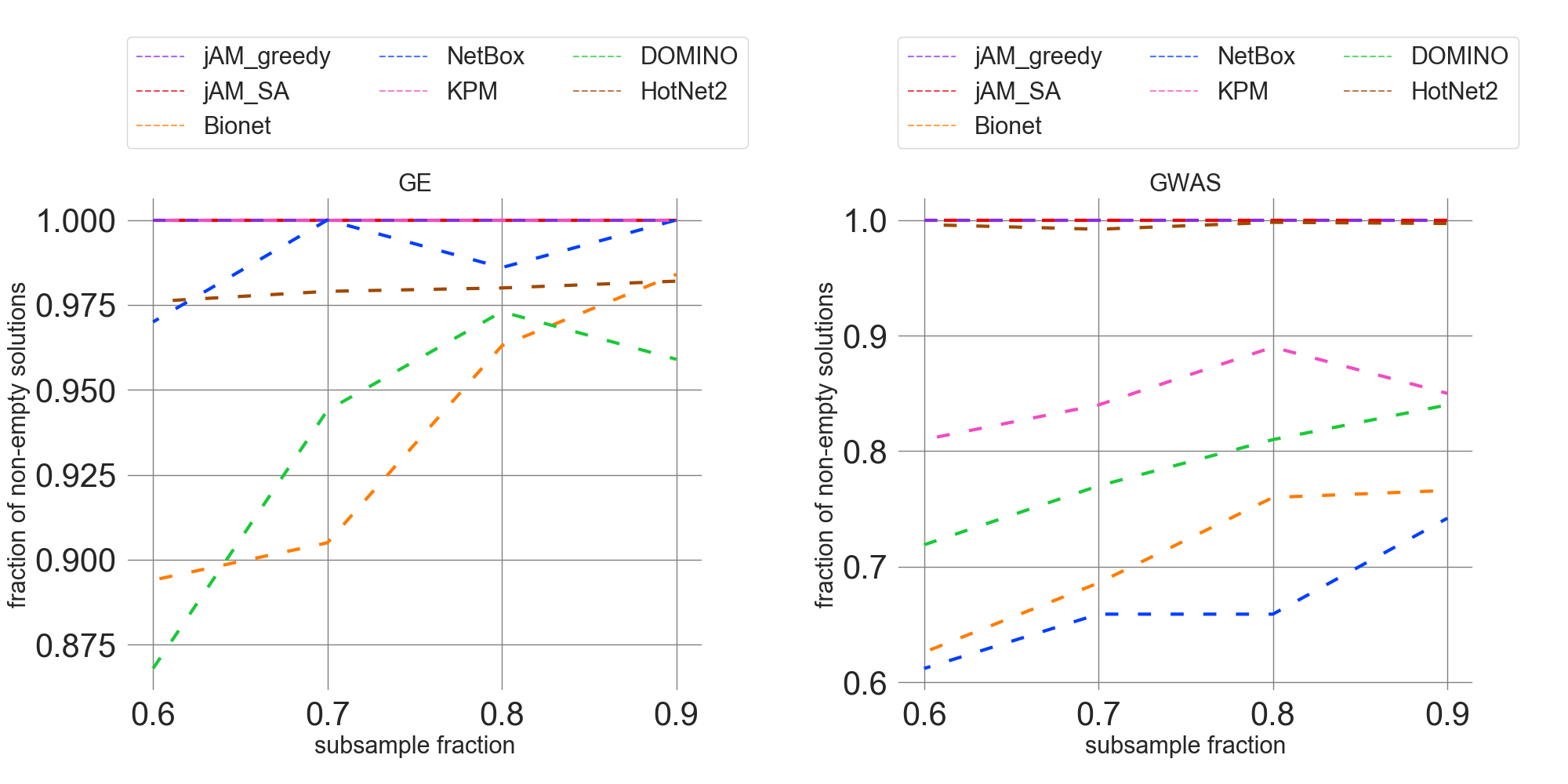


**A B**

**Appendix Figure S4**. The fraction of non-empty solutions as a function of the subsampling fraction. For each algorithm and subsampling fraction we report the average over the datasets.


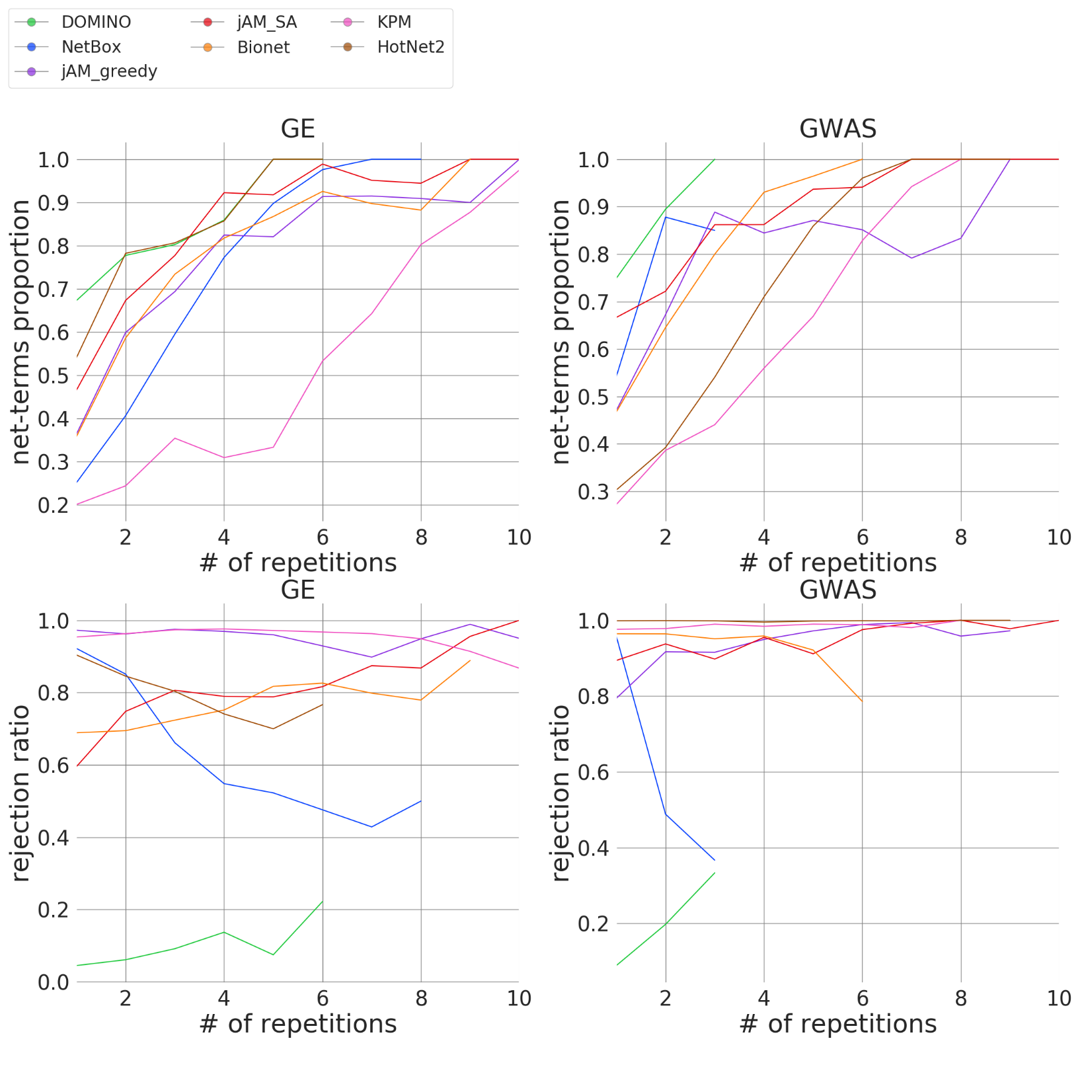


**A B**

**C D**

**Appendix Figure S5**. Comparison of the GO terms identified by each benchmarked algorithm to those identified by using the network only (net-terms). **A-B.** Proportion of net-terms as a function of the number of times the term was reported. A: GE; B: GWAS. **C-D.** Rejection ratio as a function of the number of times the term was reported. C: GE; D: GWAS.


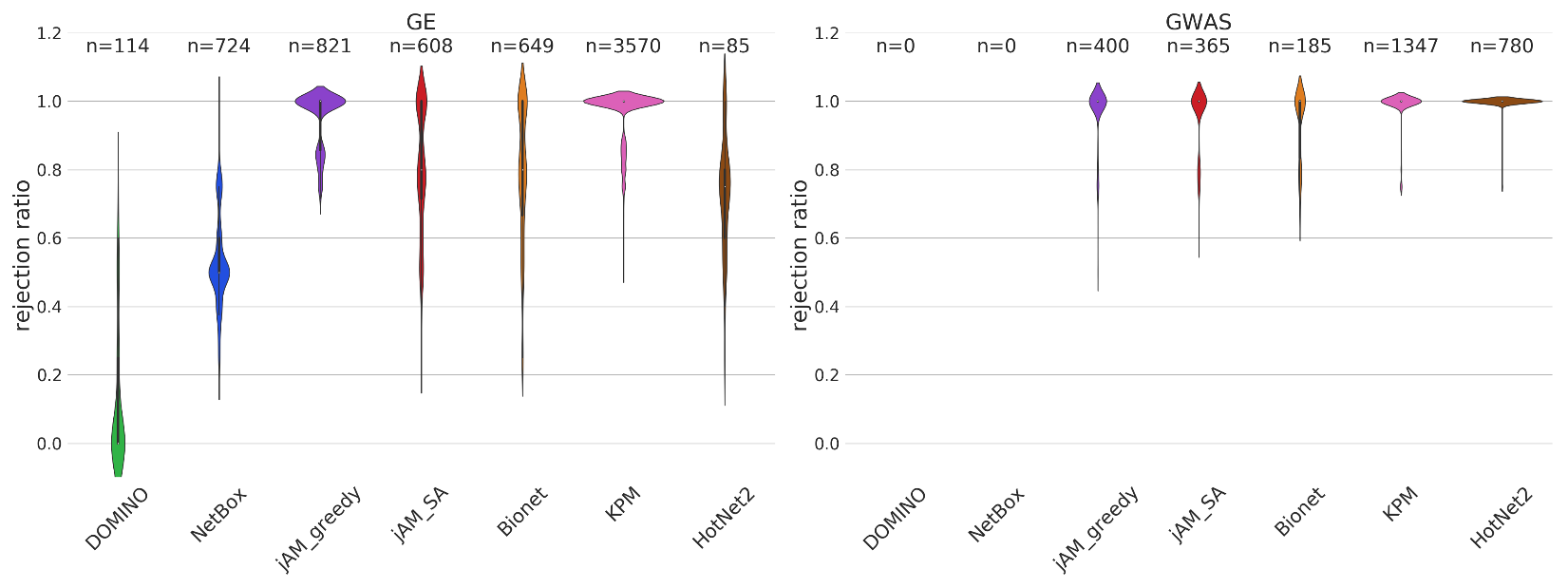


**A B**

**Appendix Figure S6**. Comparison of rejection ratios of GO terms across algorithms. **A-B**. Violin plots of rejection ratios of each algorithm in GE(A) and GWAS (B) datasets. Only terms reported in four datasets or more were included. n indicates the number of such terms. Note that no terms were reported in more than four GWAS datasets by DOMINO and NetBox, which obtained the best overall results in our benchmark.

**References**

Allen HL (2010) Hundreds of variants clustered in genomic loci and biological pathways affect human height. *Nature* **467:** 832–838

Baumbach J, Friedrich T, Kötzing T, Krohmer A, Müller J & Pauling J (2012) Efficient algorithms for extracting biological key pathways with global constraints. In *Proceedings of the genetic and evolutionary computation conference, GECCO 2012* pp 169–176.

Bayerlová M, Menck K, Klemm F, Wolff A, Pukrop T, Binder C, Beißbarth T & Bleckmann A (2017) Ror2 signaling and its relevance in breast cancer progression. *Front. Oncol.* **7:** 135

Beisser D, Klau GW, Dandekar T, Müller T & Dittrich MT (2010) BioNet: An R-Package for the functional analysis of biological networks. *Bioinformatics* **26:** 1129–1130

Cerami E, Demir E, Schultz N, Taylor BS & Sander C (2010) Automated network analysis identifies core pathways in glioblastoma. *PLoS One* **5:** e8918

Choobdar S, Ahsen ME, Crawford J, Tomasoni M, Fang T, Lamparter D, Lin J, Hescott B, Hu X, Mercer J, Natoli T, Narayan R, Aicheler F, Amoroso N, Arenas A, Azhagesan K, Baker A, Banf M, Batzoglou S, Baudot A, et al (2019) Assessment of network module identification across complex diseases. *Nat. Methods* **16:** 843–852

Connelly KE, Weaver TM, Alpsoy A, Gu BX, Musselman CA & Dykhuizen EC (2019) Engagement of DNA and H3K27me3 by the CBX8 chromodomain drives chromatin association. *Nucleic Acids Res.* **47:** 2289–2305

Elkon R, Milon B, Morrison L, Shah M, Vijayakumar S, Racherla M, Leitch CC, Silipino L, Hadi S, Weiss-Gayet M, Barras E, Schmid CD, Ait-Lounis A, Barnes A, Song Y, Eisenman DJ, Eliyahu E, Frolenkov GI, Strome SE, Durand B, et al (2015) RFX transcription factors are essential for hearing in mice. *Nat. Commun.* **6:** 593

Forbes TA, Howden SE, Lawlor K, Phipson B, Maksimovic J, Hale L, Wilson S, Quinlan C, Ho G, Holman K, Bennetts B, Crawford J, Trnka P, Oshlack A, Patel C, Mallett A, Simons C & Little MH (2018) Patient-iPSC-derived kidney organoids show functional validation of a ciliopathic renal phenotype and reveal underlying pathogenetic mechanisms. *Am. J. Hum. Genet.* **102:** 816–831

Fritsche LG, Igl W, Bailey JNC, Grassmann F, Sengupta S, Bragg-Gresham JL, Burdon KP, Hebbring SJ, Wen C, Gorski M, Kim IK, Cho D, Zack D, Souied E, Scholl HPN, Bala E, ELee K, Hunter DJ, Sardell RJ, Mitchell P, et al (2016) A large genome-wide association study of age-related macular degeneration highlights contributions of rare and common variants. *Nat. Genet.* **48:** 134–143

Hertzano R, Elkon R, Kurima K, Morrisson A, Chan SL, Sallin M, Biedlingmaier A, Darling DS, Griffith AJ, Eisenman DJ & Strome SE (2011) Cell type-specific transcriptome analysis reveals a major role for Zeb1 and miR-200b in mouse inner ear morphogenesis. *PLoS Genet.* **7:** e1002309

Ideker T, Ozier O, Schwikowski B & Siegel AF (2002) Discovering regulatory and signalling circuits in molecular interaction networks. *Bioinformatics* **18:** S233–S240

Ito T, Teo YV, Evans SA, Neretti N & Sedivy JM (2018) Regulation of cellular senescence by polycomb chromatin modifiers through distinct DNA damage- and histone methylation-dependent pathways. *Cell Rep.* **22:** 3480–3492

Kemp JP, Morris JA, Medina-Gomez C, Forgetta V, Warrington NM, Youlten SE, Zheng J, Gregson CL, Grundberg E, Trajanoska K, Logan JG, Pollard AS, Sparkes PC, Ghirardello EJ, Allen R, Leitch VD, Butterfield NC, Komla-Ebri D, Adoum AT, Curry KF, et al (2017) Identification of 153 new loci associated with heel bone mineral density and functional involvement of GPC6 in osteoporosis. *Nat. Genet.* **49:** 1468–1475

Kroeger H, Grimsey N, Paxman R, Chiang WC, Plate L, Jones Y, Shaw PX, Trejo JA, Tsang SH, Powers E, Kelly JW, Luke Wiseman R & Lin JH (2018) The unfolded protein response regulator ATF6 promotes mesodermal differentiation. *Sci. Signal.* **11:** eaan5785

De Lange KM, Moutsianas L, Lee JC, Lamb CA, Luo Y, Kennedy NA, Jostins L, Rice DL, Gutierrez-Achury J, Ji SG, Heap G, Nimmo ER, Edwards C, Henderson P, Mowat C, Sanderson J, Satsangi J, Simmons A, Wilson DC, Tremelling M, et al (2017) Genome-wide association study implicates immune activation of multiple integrin genes in inflammatory bowel disease. *Nat. Genet.* **49:** 256–261

Leiserson MDM, Vandin F, Wu H-T, Dobson JR, Eldridge J V, Thomas JL, Papoutsaki A, Kim Y, Niu B, McLellan M, Lawrence MS, Gonzalez-Perez A, Tamborero D, Cheng Y, Ryslik GA, Lopez-Bigas N, Getz G, Ding L & Raphael BJ (2015) Pan-cancer network analysis identifies combinations of rare somatic mutations across pathways and protein complexes. *Nat. Genet.* **47:** 106–14

Mahajan A, Taliun D, Thurner M, Robertson NR, Torres JM, Rayner NW, Payne AJ, Steinthorsdottir V, Scott RA, Grarup N, Cook JP, Schmidt EM, Wuttke M, Sarnowski C, Mägi R, Nano J, Gieger C, Trompet S, Lecoeur C, Preuss MH, et al (2018) Fine-mapping type 2 diabetes loci to single-variant resolution using high-density imputation and islet-specific epigenome maps. *Nat. Genet.* **50:** 1505–1513

Miano V, Ferrero G, Rosti V, Manitta E, Elhasnaoui J, Basile G & De Bortoli M (2018) Luminal lncRNAs regulation by ERα-controlled enhancers in a ligand-independent manner in breast cancer cells. *Int. J. Mol. Sci.* **19:** 593

Michailidou K, Lindström S, Dennis J, Beesley J, Hui S, Kar S, Lemaçon A, Soucy P, Glubb D, Rostamianfar A, Bolla MK, Wang Q, Tyrer J, Dicks E, Lee A, Wang Z, Allen J, Keeman R, Eilber U, French JD, et al (2017) Association analysis identifies 65 new breast cancer risk loci. *Nature* **551:** 92–94

Nelson CP, Goel A, Butterworth AS, Kanoni S, Webb TR, Marouli E, Zeng L, Ntalla I, Lai FY, Hopewell JC, Giannakopoulou O, Jiang T, Hamby SE, Di Angelantonio E, Assimes TL, Bottinger EP, Chambers JC, Clarke R, Palmer CNA, Cubbon RM, et al (2017) Association analyses based on false discovery rate implicate new loci for coronary artery disease. *Nat. Genet.* **49:** 1385–1391

Nielsen JB, Thorolfsdottir RB, Fritsche LG, Zhou W, Skov MW, Graham SE, Herron TJ, McCarthy S, Schmidt EM, Sveinbjornsson G, Surakka I, Mathis MR, Yamazaki M, Crawford RD, Gabrielsen ME, Skogholt AH, Holmen OL, Lin M, Wolford BN, Dey R, et al (2018) Biobank-driven genomic discovery yields new insight into atrial fibrillation biology. *Nat. Genet.* **50:** 1234–1239

Pulikkan JA, Hegde M, Ahmad HM, Belaghzal H, Illendula A, Yu J, O’Hagan K, Ou J, Muller-Tidow C, Wolfe SA, Zhu LJ, Dekker J, Bushweller JH & Castilla LH (2018) CBFβ-SMMHC inhibition triggers apoptosis by disrupting MYC chromatin dynamics in acute myeloid leukemia. *Cell* **174:** 172-186.e21

Ripke S, Neale BM, Corvin A, Walters JTR, Farh KH, Holmans PA, Lee P, Bulik-Sullivan B, Collier DA, Huang H, Pers TH, Agartz I, Agerbo E, Albus M, Alexander M, Amin F, Bacanu SA, Begemann M, Belliveau RA, Bene J, et al (2014) Biological insights from 108 schizophrenia-associated genetic loci. *Nature* **511:** 421–427

Schmidt SF, Larsen BD, Loft A, Nielsen R, Madsen JGS & Mandrup S (2015) Acute TNF-induced repression of cell identity genes is mediated by NFκB-directed redistribution of cofactors from super-enhancers. *Genome Res.* **25:** 1281–1294

Shannon P, Markiel A, Ozier O, Baliga NS, Wang JT, Ramage D, Amin N, Schwikowski B & Ideker T (2003) Cytoscape: A software environment for integrated models of biomolecular interaction networks. *Genome Res.* **13:** 2498–2504

Teslovich TM, Musunuru K, Smith A V., Edmondson AC, Stylianou IM, Koseki M, Pirruccello JP, Ripatti S, Chasman DI, Willer CJ, Johansen CT, Fouchier SW, Isaacs A, Peloso GM, Barbalic M, Ricketts SL, Bis JC, Aulchenko YS, Thorleifsson G, Feitosa MF, et al (2010) Biological, clinical and population relevance of 95 loci for blood lipids. *Nature* **466:** 707–713
