# Supplementary material for: DOMINO: a network‐based active module identification algorithm with reduced rate of false calls
Source: Mol Syst Biol. 2021 Jan 20;17(1):e9593. doi: 10.15252/msb.20209593 (PMC7816759; doi:10.15252/msb.20209593)
Supplement: Supplementary file 2 — Expanded View Figures PDF [file MSB-17-e9593-s002.pdf]

Expanded View Figures

Figure EV1. Module-level EHR (mEHR) scores on the GWAS datasets for each algorithm and GWAS dataset.
Up to ten top modules are shown per dataset, ranked by their mEHR. Dot size represents module size. The EHR column in green shows the number of EV terms and the number of significant terms found.

© 2021 The Authors

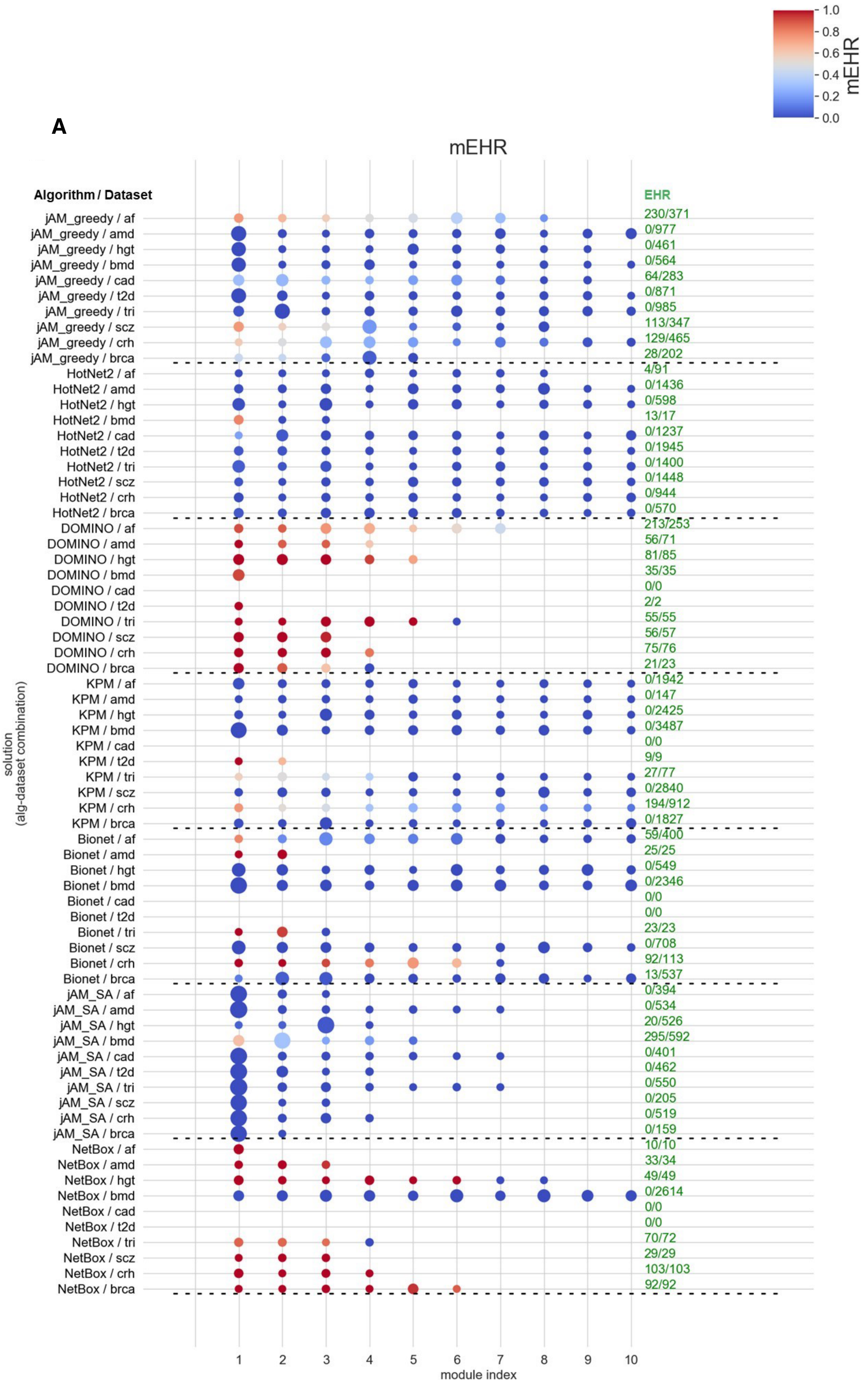

Figure EV1.

© 2021 The Authors

Figure EV2. Evaluation results for the GWAS datasets.

A Module-level EHR scores. The plots show average mEHR score in the k top modules, as a function of k. Modules are ranked by their mEHR scores.
B Biological richness. The plots show the median number of non-redundant terms (richness score) as a function of the Resnik similarity cutoff.
C Intra-module homogeneity scores as a function of Resnik similarity cutoff.
D Robustness measured by the average AUPR over the datasets, shown as a function of the subsampling fraction.
E Robustness measured by the average F1 over the datasets shown as a function of subsample fraction (results for each dataset and fraction were averaged over 100 subsampling).

© 2021 The Authors

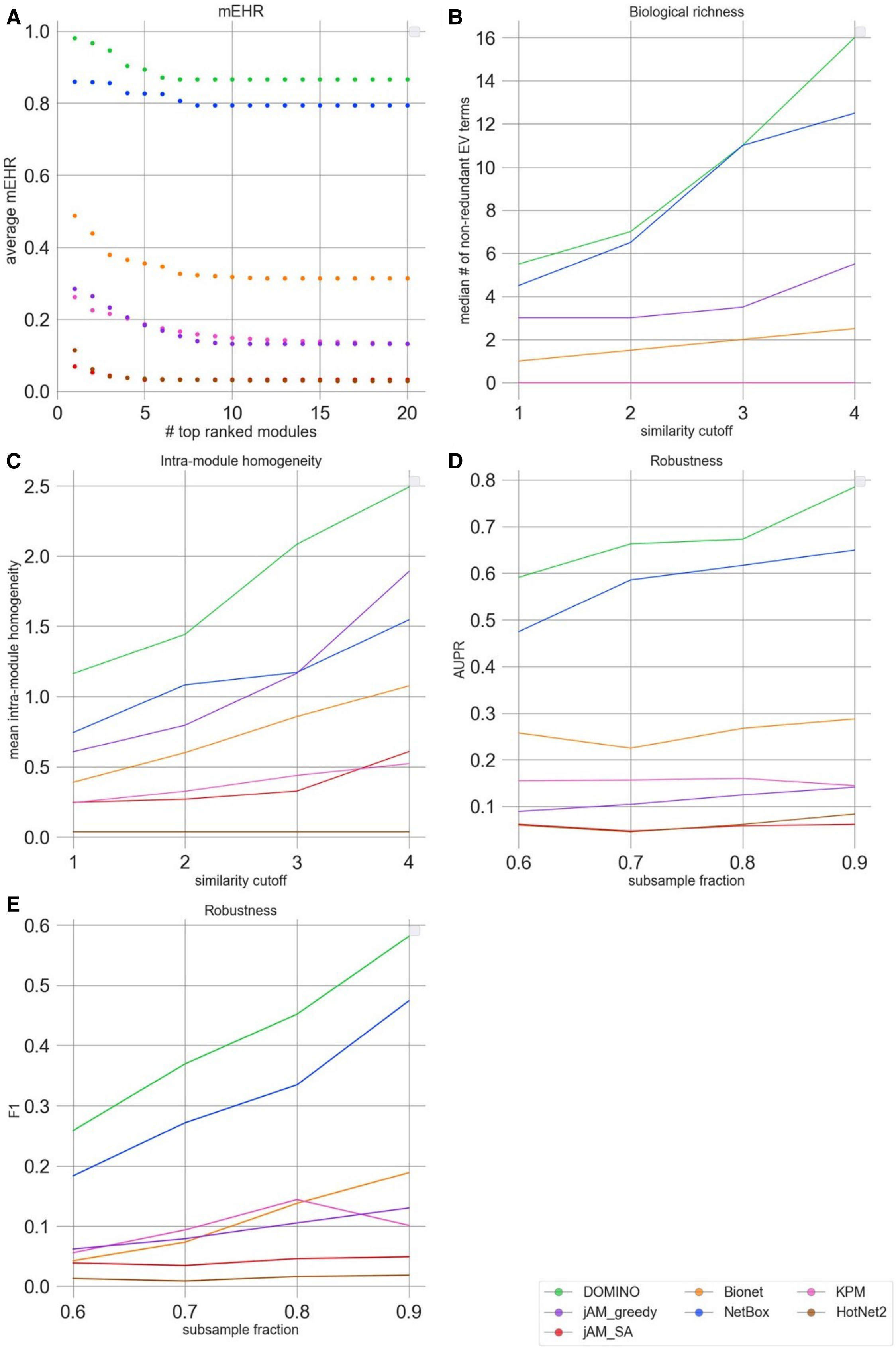

Figure EV2.

 © 2021 The Authors
